# Unraveling the Dynamic Properties of New-Age Energy Materials Chemistry Using Advanced In Situ Transmission Electron Microscopy

**DOI:** 10.3390/molecules29184411

**Published:** 2024-09-17

**Authors:** Subramaniyan Ramasundaram, Sampathkumar Jeevanandham, Natarajan Vijay, Sivasubramani Divya, Peter Jerome, Tae Hwan Oh

**Affiliations:** 1 School of Chemical Engineering, Yeungnam University, Gyeongsan 38541, Republic of Korea; ramasundaram79@hotmail.com (S.R.); jmcvijay@gmail.com (N.V.); divi.fysics@gmail.com (S.D.); jeromepeter7688@gmail.com (P.J.); 2 Molecular Science and Engineering Laboratory, Amity Institute of Click Chemistry Research and Studies, Amity University, Noida 201313, Uttar Pradesh, India; sampathkumarj4@gmail.com

**Keywords:** energy storage, energy conversion, in situ TEM, cryo-TEM, FIB, electrochemistry

## Abstract

The field of energy storage and conversion materials has witnessed transformative advancements owing to the integration of advanced in situ characterization techniques. Among them, numerous real-time characterization techniques, especially in situ transmission electron microscopy (TEM)/scanning TEM (STEM) have tremendously increased the atomic-level understanding of the minute transition states in energy materials during electrochemical processes. Advanced forms of in situ/operando TEM and STEM microscopic techniques also provide incredible insights into material phenomena at the finest scale and aid to monitor phase transformations and degradation mechanisms in lithium-ion batteries. Notably, the solid–electrolyte interface (SEI) is one the most significant factors that associated with the performance of rechargeable batteries. The SEI critically controls the electrochemical reactions occur at the electrode–electrolyte interface. Intricate chemical reactions in energy materials interfaces can be effectively monitored using temperature-sensitive in situ STEM techniques, deciphering the reaction mechanisms prevailing in the degradation pathways of energy materials with nano- to micrometer-scale spatial resolution. Further, the advent of cryogenic (Cryo)-TEM has enhanced these studies by preserving the native state of sensitive materials. Cryo-TEM also allows the observation of metastable phases and reaction intermediates that are otherwise challenging to capture. Along with these sophisticated techniques, Focused ion beam (FIB) induction has also been instrumental in preparing site-specific cross-sectional samples, facilitating the high-resolution analysis of interfaces and layers within energy devices. The holistic integration of these advanced characterization techniques provides a comprehensive understanding of the dynamic changes in energy materials. This review highlights the recent progress in employing state-of-the-art characterization techniques such as in situ TEM, STEM, Cryo-TEM, and FIB for detailed investigation into the structural and chemical dynamics of energy storage and conversion materials.

## 1. Introduction

The development of new-age energy materials is at the forefront of scientific research, driving numerous advancements in the field of energy storage and conversion technologies including metal rechargeable batteries, fuel cells, perovskites, photocatalysts, etc. [1,2,3,4,5,6,7,8,9,10,11]. Transmission electron microscopy (TEM) is a powerful technique used to visualize materials at the atomic scale with the highest resolution possible. The advent of nanotechnology requires more understanding of the real-time changes in the structure and physicochemical properties of functional materials. Even though TEM reveals physicochemical information such as structure and composition at nanoscale and subsecond resolution, conventional TEM is not suitable for the real-time study of materials used in energy storage systems. Real-time monitoring systems must be operated in an environment that is often manipulated by external factors such as heat, gas, liquid, electric and magnetic fields, etc. The insights gained from real-time analysis are useful for diagnosing the issues and attaining solutions. Functional materials with improved properties can be delivered by addressing the diagnosed issues. Advancements in TEM led to the development of tools for in situ analysis capable of rendering information that is inaccessible by conventional ex situ analysis. In in situ TEM studies, the results have been collected dynamically as a function of changes in synthesis conditions, and the effect of various stimuli (heat, voltage, environment, force, mechanical stress, magnetic field, and light) [9,11,12,13]. The tools to duplicate the identical synthesis conditions and the application of various stimuli are installed inside the sample compartment. Sample holders have been adequately modified to accommodate the stimuli sources and testing environment. In situ TEM techniques render an immeasurable depth of information about nanomaterials. In situ TEM-drawn real-time observation analysis replicates the benchtop reaction conditions, increases the accuracy of data, rectifies scientific misconceptions, provides solutions for various technical issues, and favors unexplored discoveries. To meet the growing energy demands, a variety of functional materials have been developed for energy conversion (heterogeneous catalysts and perovskites) and storage (batteries and supercapacitors) applications. In situ TEM finds tremendous application in the development of next-generation energy materials. As the practical operation (operando) environment can be imitated, in situ TEM studies provide direct evidence for structural changes relevant to stability, efficiency, and safety [13,14].

In batteries, a rise in localized temperature under operating conditions causes serious concern. Heat increase is a ubiquitous phenomenon that triggers material degradation, disrupts the energy supply, and may lead to dangerous safety issues. In the nanoscale, electrode materials display inhomogeneous behavior. Thus, characterization tools suited for individual particle-level evaluation are necessary to draw detailed mechanisms in their operating environments. To understand and overcome these issues, in situ TEM studies are performed with appropriate thermal stimuli. In lithium-ion batteries (LiBs), lithiation of the electrode occurs during the charge/discharge cycle. Lithiation is known to induce several detrimental changes to the electrode including swelling, fracture, disintegration, change of crystalline phases, alterations in overall crystallinity, and intermediate-mediated changes in charging rate. Lithiation-induced changes led to capacity fading and failure [15]. In situ TEM allows the creation of a single nano electrochemical cell, and biasing (voltage application). At the level of a single nanowire or particle-level monitoring, the mechanical behaviors (bending, breakage, and disintegration) can be evaluated in detail. Such in-depth observations provide a route for obtaining high-performance electrode materials with optimized morphological features (length, diameter, and capping with protective material). Heterogenous catalysts are used for energy production via thermal catalytic conversion and photocatalytic hydrogen evolution processes [2,5,16]. Thermal catalysts such as Ni/TiO_2_ nanoparticles (NPs) are being used for converting CO_2_ to methane and other useful chemicals via high-temperature catalytic hydrogenation [17]. In situ TEM studies can be used to monitor the condition at which suboxide overlayer formation occurs on Ni. Suboxide overlayer blocks the catalytically active surface area. In situ TEM equipped with light stimuli is used for observing the origin of H_2_ evolution on the surface of the catalyst. Perovskites are most commonly used for solar energy conversion. However, they easily undergo degradation in the presence of air, moisture, and heat. Using in situ TEM studies enables the identification of conditions that cause degradation when exposed to air, inert gas atmosphere, light, and heat.

**Scheme 1:**
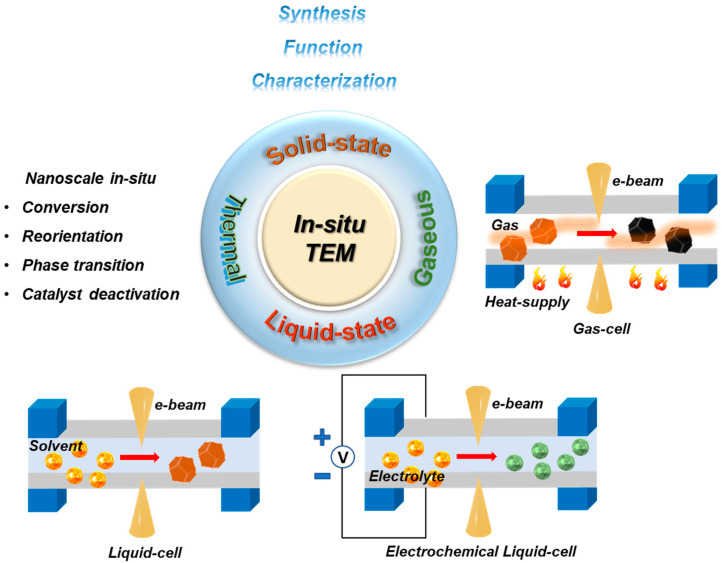
Overview of diverse methodologies adapted for in situ TEM characterization of energy materials.

TEM combines imaging, electron diffraction, and spectroscopic tools and provides valuable information about morphology, crystalline structure, and chemical composition of materials [18]. As required for the aforementioned reasons, a suitable TEM tool was selected among various techniques available. Environmental TEM (ETEM) is used to study the gas–solid interface [6]. Atomically precise data construction replicating benchtop conditions can be obtained from spherical aberration-corrected TEM (Cs-TEM) [19]. Electron energy loss spectroscopy (EELS) is used to obtain spatially resolved spectra and direct visualization of structures. Under applied stimuli, scanning TEM (STEM) is employed for obtaining changes in particle size, crystalline structure, and surface defects [20]. Generally, the high-energy electron beams used in TEM measurements often degrade materials with low stability, weak bonds, and interfaces. In such conditions, cryogenic TEM (Cryo-TEM) techniques are more suitable for the detailed investigation of materials with fragile composition. Cryo-TEM protects the sample from radiation and the environment is usually maintained under ultra-low temperature conditions. In situ Cryo-TEM is used for obtaining atomic-scale structural and chemical properties of battery materials and perovskites [21]. Focused ion beam (FIB) milling in TEM is one of the finest techniques which possess the ability to examine specific regions of interest with high-resolution characterization and to obtain site-specific electron diffraction patterns. The current review discusses recent efforts made towards using the above in situ TEM studies for the development of next-generation energy storage and conversion materials. The focus is placed on studies performed using thermal, gaseous, environmental, photovoltaic, and mechanical stimuli. Scheme 1 illustrates the comprehensive representation of various in situ TEM-based analyzes meant for nanoscale/atomic-level characterization of energy materials.

## 2. In Situ Solid-State and Electrochemical Biasing Integrated TEM Characterization for Energy Materials

Maintaining the thermal stability of solid-state energy materials under real-time reaction conditions in numerous catalytic processes has always been a crucial concern over the past few decades in lab-scale and industrial applications [22,23,24,25,26,27]. Such a phenomenon is usually caused in nanostructured energy materials, which often undergo drastic transitions in their intrinsic compositions and surface structures when externally influenced by thermal or electric/magnetic-based stimuli. Hence, an extensive understanding of the intermediate reactions is necessary for designing rational materials suitable for extreme operating conditions. Some commonly used functions, particularly the in situ heating tools in TEM, have undergone diverse changes. Sample holders encountered several limitations including thermal stability at temperatures above 1000 °C. This is one of the prominent issues observed with the furnace-type or spiral-shaped tungsten wire-based holders [28,29,30]. Sample assemblies with uneven temperature distribution, drastic thermal drift, and strong X-ray shadowing effects are determinantal for atomic-scale in situ characterization. To overcome these drawbacks, in situ TEM equipped with advanced microelectromechanical systems (MEMSs) or electrochemical characterization tools was developed. These tools enable localized heating with nanopatterned surfaces and offer precise control over temperatures ranging from 1100–1300 °C [30]. Also, these tools facilitate reaching desired conditions rapidly within milliseconds, tremendously minimize the specimen drift due to heat stress, and allow fast stabilization of nanostructured materials during in situ observations. Mostly, lithium-ion-based energy materials with lithiation/de-lithiation chemistry often require in situ heat-induced observations at the nanoscale to precisely understand the innately complex mechanisms under operating conditions.

To establish a comprehensive understanding of the lithiation of Si electrodes, McDowell et al. used in situ TEM [31]. Their effort was directed towards obtaining clarity and understanding the mechanistic behavior of dynamic aspects that influence the lithiation of Si electrodes, Li diffusion, rate of reaction at different surfaces, and mechanical stress. They performed real-time monitoring of lithiation effects in crystalline Si NPs, observing the time dependence of the mechanical stress induced by lithiation, and analyzed overall reaction kinetics. For in situ TEM experiments, a nanoscale electrochemical cell was assembled in the following manner. Si nanowire (NW) coated with a ~30 nm layer of Cu on a sidewall was attached to the bottom of the TEM holder. A cluster of Si NPs was then attached to Si nanowires (NWs). On top of the holder, the powder electrode (LiCoO_2_) and ionic liquid (IL) electrolyte were coated. To this electrochemical cell, electrical biasing was applied to the Si electrode by applying −3.7 and −4.0 V. The study was performed at the level of individual particles. Progression of lithiation was slow in the front position. As the reaction proceeded, changes occurred in the mechanical stress at the lithiation front which altered the overall driving force and controlled the reaction to proceed slowly. An extreme volume change occurred at the interface of crystalline and lithiated Si and caused large stress. The shape of the particle dictated the stress evolution. The observed phenomenon was reported to be unique to crystalline Si and is not correlated with the phase transformations observed in conventional materials that experience much smaller stresses and strains. The stresses and strains developed during lithiation/de-lithiation of amorphous Si were expected to be very different from crystalline Si. Both in crystalline and amorphous Si, the effect of particle morphology, reaction thermodynamics, stress, diffusion, and kinetics were found to be interrelated. These findings were expected to contribute to the development of Si electrodes with optimum morphology and cyclic stability.

**Figure 1:**
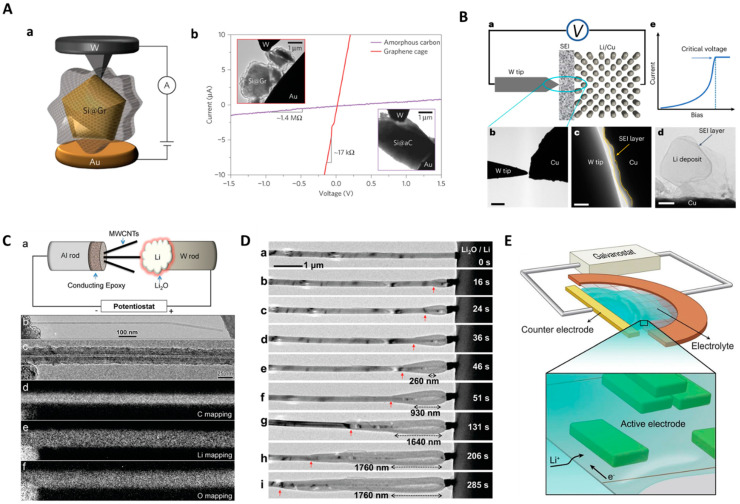
In situ solid-state/electrochemical biasing TEM characterization for energy materials. (**A**) Single-particle-level characterization of (**a**) graphene cage-like layer covering connected with the electrical circuit for external load test analysis, and (**b**) current-voltage measurements of graphene-encapsulated and amorphous-carbon-coated SiMPs [32]. (**B**) Experimental set-up of (**a**) in situ bias arrangements of tungsten tip and Cu wire inside TEM, (**b**) low-magnification TEM showing the contact, (**c**) high-magnification TEM showing the contact between tip, wire, and SEI layer, and (**d**) TEM showing the surface SEI layer assembly of the integrated set-up, and (**e**) current-voltage plot indicating critical voltage [33]. (**C**) Lithiation of electrochemically biased and (**a**) arc-discharged MWCNT which is glued to an Al rod (working electrode), Li_2_O grown on Li surface acts as a solid electrolyte, bulk Li metal scratched from tungsten rod acts as counter electrode, (**b**) pristine MWCNT before coming in contact with Li_2_O/Li electrode, (**c**) lithiated MWCNT showing uniform Li_2_O layer formation on the surface, and (**d**–**f**) corresponding EELS mapping of C, Li, and O, respectively, indicating the nanotube lithiation [34]. (**D**) Crack formation in the lithiated Si NWs, (**a**–**i**) morphological evolution showing anisotropic elongation and crack during the lithiation of solid cells after contacting the Li_2_O/Li electrode. Red arrows in the panel indicate the propagation of reaction front [38]. (**E**) In situ electrochemical functional cell for operando TEM characterization of battery materials [41]. Figures reproduced with permission from [32,33,34,38,41].

Li et al. used in situ TEM to study the lithiation properties of graphene cage (GC)-encapsulated Si microparticles (SiMPs) (Figure 1A) [32]. GC encapsulation was carried out to protect the mechanical stability of SiMPs during lithiation. As mentioned in previous reports, Si particles with a diameter > 150 nm and Si NWs with a length > 250 nm experienced lithiation-induced fracture, broke into pieces, and led to eventual failure of the electrochemical cell. GC encapsulation was carried out by conformal growth on SiMPs. To create a cell, in the TEM holder, Li metal-coated tungsten wire and GC-encapsulated SiMPs were brought into contact by the piezo manipulator. To initiate the lithiation, a bias of −3 V was applied to the Li metal with native Li_2_O (solid electrolyte). During lithiation, the GC acted as a mechanically flexible and strong buffer. The GC buffer layer allowed the expansion and fracture of SiMPs inside the cage. Due to the GC protecting layer, the electrical properties of SiMPs are retained at the scale of particles and electrodes. In situ TEM studies indicated that a stable solid–electrolyte interface formed by a GC minimized the irreversible consumption of Li ions and rapidly increased Coulombic efficiency during initial cycling. In this study, in situ TEM was effectively utilized to monitor the lithiation properties of SiMPs and the development of stable protecting layers. Further, this study resolved the two long-standing issues of SiMPs (fracture and stability of the solid–electrolyte interface). Further, in situ TEM studies can be opted for developing other protection layers for SiMPs, electrode materials, and the development of stable and inexpensive batteries.

Recently, Xu et al. discussed an innovative study on the characterization of the solid–electrolyte interphase (SEI) in rechargeable batteries using in situ transmission electron microscopy (TEM) [33]. The SEI is a critical component that significantly influences battery performance, and understanding its electrical properties is crucial for improving battery technology. They used in situ bias TEM integrated with scanning tunneling microscopy (STM) to directly measure SEI electrical properties (Figure 1B), and employed a tungsten nanoprobe as a counter electrode. Measurements were conducted at low magnification to avoid electron beam damage, and to complement the experiments, ab initio molecular dynamics (AIMD) simulations were also performed. Four types of electrolytes were examined, including low-concentration (LCE), high-concentration (HCE), localized high-concentration (LHCE), and pseudo-localized high-concentration (PLHCE) electrolytes. These electrolytes were based on lithium bis(fluorosulfonyl)imide (LiFSI) and 1,2-dimethoxyethane (DME) with various additives. SEIs exhibit voltage-dependent differential conductance, contrary to the common assumption of them being purely insulating. Higher differential conductance rates correlate with thicker SEIs and more complex topographic features. These characteristics lead to inferior Coulombic efficiency and cycling stability in battery cells. The study provides insights into the electrical nature of SEIs, which is crucial for designing better-performing batteries. It challenges the conventional view of SEIs as purely insulating layers. These findings suggest that tuning the electrical properties of SEIs could be a pathway to enhancing battery performance. Previously, direct measurement of SEI electrical properties was considered a challenging task to achieve. A combination of experimental techniques (in situ TEM and STM) with computational modeling (AIMD) was used for the systematic study of different electrolyte compositions, and to understand their impact on SEI formation and properties. The measurement setup using two blocking electrodes provides an upper limit of electrical conductivity rather than an exact replication of a real battery system. This study represents a significant advancement towards understanding the electrical properties of SEIs in rechargeable batteries. By providing direct measurements and revealing the voltage-dependent conductance of SEIs, it challenges existing assumptions and opens new avenues for battery design and optimization. The insights gained from this research could lead to the development of SEIs with tailored electrical properties, potentially improving the overall performance and longevity of rechargeable batteries.

In a work by Liu et al., an Al rod attached to multiwalled CNTs (MWCNTs) was brought into contact with Li bulk metal scratches on tungsten. The solid electrolyte, Li_2_O was allowed form on the Li scratches by exposing to air for 2 s. The voltaic cell was formed when both came into contact with a piezo manipulator (Figure 1C) [34]. Lithiation was initiated by applying the potential of −2 V to the MWCNTs part. The MWCNTs part was visualized by the image obtained using safer irradiation doses so that no irradiation-induced mechanical defects occurred. To measure the mechanical response, the MWCNTs part was pushed and pulled towards the surface of the Li_2_O/Li electrode. An individual MWCNT was monitored. Liu et al. found a 0.4 Å increase in innertube spacing (3.4 to 3.6 Å), which corresponds to 5.9% circumferential and radial expansion. On the outermost wall of the MWCNT, the tensile hoop stress was measured to be about ~50 GPa. Upon lithiation, the straight walls of the tube were sharp-edged, and brittleness with fracturing was also noticed upon an in situ test meant for compression and tension. The authors indicated that this is in sharp contrast with the extremely flexible pristine MWCNTs; upon tension, they fail in a “sword-in-sheath” manner. Stretching C–C bonds, strain, and electron transfer to the antibonding π orbital were attributed to the lithiation-induced mechanical fracture of MWCNTs. Further, quantum chemical modeling revealed that the combination of tensile hoop prestress, mechanical weakening, and stretching of C–C bonds by Li^+^ are the reasons for the lithiation-induced disintegration of MWNTS.

Si (Li_15_Si_4_) possesses the highest specific capacity (~3579 mAh/g) among all other anodes preferred for LiBs. However, the volume expansion of Si anodes during lithiation is large (~300%). This led to huge capacity loss and pulverization in the first lithiation cycle [35,36,37]. Liu et al. employed in situ TEM studies to understand the fracture mechanisms and explore the strategies for mitigating the volume expansion and degradation of mechanical properties [38]. Using conducting epoxy, a small piece of Si wafer with Si nanowires (Si NWs) was attached to an Al rod. Tungsten wire with Li metal adhered was prepared by the scratching method. Both Si and Li electrodes were mounted on the TEM holder. The electrolytes used were air-grown solid Li_2_O or IL. In an IL electrolyte (ILE)-filled cell, bulk LiCoO_2_ counter-electrode was used instead of Li metal. The ILs used were lithium bis (trifluoromethylsulfonyl) imide and 1-butyl-1-methylpyrrolidinium bis (trifluoromethylsulfonyl) imide. The working electrode in the solid cell was a single Si NW. Lithiation was initiated by applying potential. After Li^+^ reaches Si, the diffusion and reaction become intrinsic to the Si NWs. This type of electrolyte does not influence the diffusion kinetics of the reaction. During the first step of lithiation, anisotropic swelling of SiNWs was noticed in both solid and liquid cells. This was attributed to the crystallographic orientation-sensitive interfacial processes occurring to accommodate large volumetric strains. The cross-section of Si NWs undergoing an anisotropic swelling appeared in a dumbbell shape (Figure 1D). The reason for the dumbbell shape is tensile hoop stress-induced plastic flow and necking instability. Lithiation-induced anisotropic swelling was reported as causing morphonology instability, fracture of Si NWs, and eventually the decay of specific capacitance.

Lithium titanate (LTO) is an appealing anode considered for fast charging and it is free from the Li plating issues found in graphite-based anodes [39]. In LTO, several challenges persist in the determination of Li’s atomic configuration in Li_4+x_Ti_5_O_12_, a metastable intermediate formed during lithiation, and the associated transport pathway of Li^+^ ions. Li K-EELS (Li-EELS) is sensitive to the environment encompassed by Li and promising for determining the energy-loss near-edge structure and the site occupancy of Li. In the presence of liquid electrolyte and thick membrane, the low-lying Li K edge (~60 eV) is more prone to be affected by plural inelastic scattering and the strong plasmon excitations [40]. Thus, it makes the use of operando Li-EELS analysis complicated. To overcome the above issues, Zhang et al. created an IL-based electrochemical cell in TEM and enabled operando Li-EELS for studying the lithiation in LTO, probing Li occupancy and transport at various rates of charging [41]. A TEM grid-based electrochemical cell was created for this study. The IL used was lithium bis(trifluoromethanesulfonyl)imide in 1-butyl-1-methylpyrrolidinium bis(trifluoromethylsulfonyl) imide. The IL thickness was 1–10 nm or less. The cell was studied at a cycling rate between 0.8 to 8 C. Fast-charging batteries typically use electrodes capable of accommodating lithium continuously using solid–solution transformation because they have few kinetic barriers apart from ionic diffusion. One exception is lithium titanate (Li_4_Ti_5_O_12_), an anode exhibiting extraordinary rate capability inconsistent with its two-phase reaction and slow Li diffusion in both phases. This experiment revealed that kinetic pathways along two-phase boundaries and distorted Li polyhedra in metastable intermediates enabled facile Li titanate transport in Li_4+x_Ti_5_O_12_ (Figure 1E). The authors pointed out that assessing an energy landscape above the ground state can enable high-rate capability. Li^+^ migration routes revealed the existence of intermediate configurations in face-sharing Li polyhedra. The apparent difference between the high-rate capability and poor Li^+^ conductivity was reconciled by the effect of low Li^+^ migration barriers along with the low formation energy of the interfaces. These findings provided useful insights about accessibility at high rates, and improved kinetics originated from intermediate configurations, particularly from their face-sharing Li motifs. This study is a step further toward designing electrodes capable of fast charging.

## 3. In Situ Gas-Phase/Environmental TEM (ETEM) and Integrated Thermal TEM Characterization for Energy Materials

### 3.1. Thermal (or Heat)-Induced In Situ TEM-Based Characterization

Integrating thermal systems with an optimized reactive gas-phase environment as a miniature model in the TEM characterization replicates gas-induced chemical reactions associated with the energy materials. In this system, gas is injected into the microscope and the transformation pathways of the solid–gaseous state reaction are monitored in real time [18,42,43,44,45,46]. Some of the significant challenges in these integrated systems are maintaining ultra-high vacuum conditions and scattering of incident electrons with the injected gas molecules [47]. Though closed-gas cell holders have numerous advantages, the typical integration of heating functions reduces the spatial resolution and hampers some real-time acquisition of electron diffraction spectra due to the limited sample tilting range [48].

**Figure 2:**
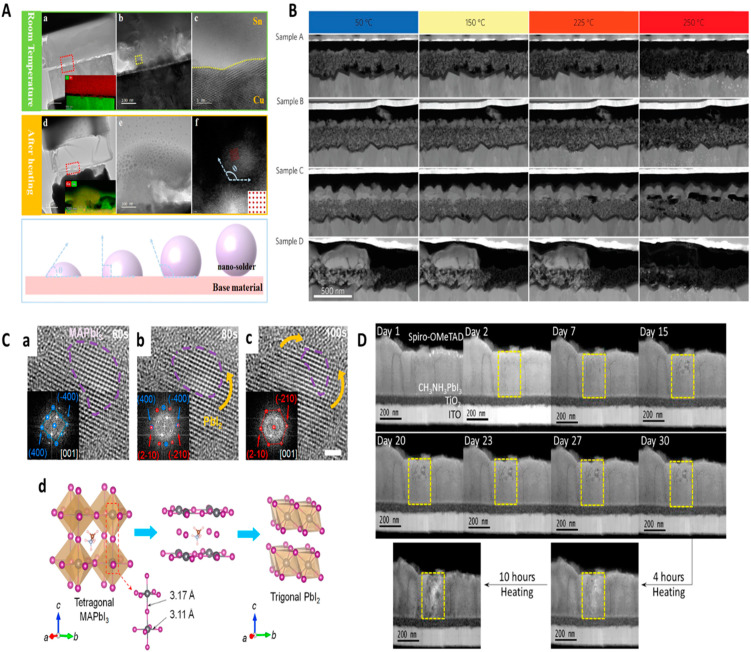
In situ thermal-induced TEM characterization of energy materials. (**A**) Thermal welding type experiment of nano solder Sn sheet and Cu structure, showing (**a**) TEM images of their interface at room temperature (Red, and green parts represent Sn, and Cu, respectively) and (**d**) after heating (enlarged version (**b**,**e**) and HRTEM images (**c**,**f**) before and after heating, respectively) [50]. (**B**) STEM-HAADF images of samples at all four conditions of the perovskite layer (varying the temperature from 50 to 250 °C) showed no visible changes until 150 °C [1]. (**C**) Direct transitions of perovskite structures from tetragonal to trigonal crystalline structure, (**a**–**c**) degradation process focusing on single MAPbI_3_ grains (scale bar = 2 nm), and (**d**) MAPbI_3_ transition from tetragonal phase to PbI_2_ with a trigonal configuration [51]. (**D**) Cross-sectional view of in situ analysis showing HAADF images of a perovskite solar cell with a device structure of glass/ITO/TiO_2_/CH_3_NH_3_PbI_3_/spiro-OMeTAD/Ag, and stability check: day 1 shows bright images due to FIB beam focus damage, day 2 shows beam-sensitiveness slowly disappeared, days 7–15 show growth of electron-beam damaged area, and days 20–30 show no further changes (later day-30 samples underwent heating-induced degradation at different time intervals as shown by arrows) [52]. Figures reproduced with permission from [1,50,51,52].

Hence, utilizing the high-resolution Cs-TEM technique is indispensable for providing sub-nanoscale spatial resolution. Generally, Cs-TEM is used to analyze morphology, physiochemical properties, and atomic occupancy. In situ Cs-TEM has been used to dynamically investigate the changes in physiochemical properties and a multitude of structural changes. For instance, monitoring the transformations of physicochemical properties and composition of anode materials in rechargeable batteries including LiBs is of greater interest. However, currently Sn-based anode materials, metal oxides, sulfides, etc. are often used in LiBs due to their low cost, high safety, eco-friendliness, and high specific capacity [49]. Zhou et al. used in situ Cs-TEM to find out the reasons that prevent the use of pure Sn as a soldering material, while Sn plays an indispensable and main component in Sn-based soldering materials used in electronic packaging (Figure 2A) [50]. By using Cs-TEM, an appropriate tool for the evaluation of microstructural changes, the authors investigated the structural changes that occurred in Sn nanosheets (NSs) under thermal field conditions. For this study, a thin sheet of Sn with a thickness of 100 nm was cut out from a single Sn crystal. The Pt perfecting layer was coated on the Sn sheet, milled using gallium ion beams, and polished into the required shape. The polished thin sheet was placed on the grid with a tungsten tip. The grid was then transferred to a heating chip. The heating process was carried out using the Protochips system. While heating, Sn nanospheres (NSPs) were formed. Continuous heat preservation led to changes in the crystal structure of the NSPs. The unstable crystal structure decreases the contact surface area and causes instability at the point of contact. These factors pose challenges when pure Sn is used as a soldering material. The morphology of the Sn–Cu interface obtained via ex situ Cs-TEM further validated this conclusion. In the Cu–Sn welding interface, Sn NSPs were observed, and their contact angle exceeded 90°. Further, it was reported that in the initial heating step, Sn NSPs underwent rapid melting and diffusion, and formed distinct Sn particles. When the heating rate was decreased, the NSPs were recrystallized into larger particles. Though the presence of NSPs increased the specific surface area, changes in crystal orientation posed challenges in welding. Notably, in the heating process, instead of forming reliable intermetallic compounds, Sn particles were formed. These findings are certainly useful to comprehend the difficulties associated with the use of pure Sn as a solder in energy devices, and similar issues.

Thermal stability is one of the most significant factors that hinder the next-generation progress of perovskite solar cell technology. Various thermal degradation mechanisms were reported in nanostructured hybrids. It is essential to explore the thermal properties of meso-superstructured perovskite composite (MSPC). In this regard, Divitini et al. introduced four different sample preparation methods, which allow the correlation of synthesis, morphology, photovoltaic characteristics, and monitoring of the behavior when heating. In this regard, Divitini et al. introduced four different sample preparation methods or conditions [1]. The four methods or conditions employed were vacuum conversion (A); conversion in glove box (B); conversion in air atmosphere with the relative humidity of 50% (C); and a single-step glove box approach (D) (Figure 2B). For this study, the authors combined several recent advances relevant to TEM. Detectors capable of fast acquisition with low electron dose, high brightness electron guns, and de-noising algorithms were employed. A highly stable-cum-fast response micro-heater-based in situ heating holder facilitating fast heating/cooling was also used for the analysis. Samples were heated up to 250 °C. The morphology of samples B and C exhibited perovskite infiltration within titanium and a homogeneous capping layer. Incomplete infiltration and a very thin capping layer were noticed in Sample A. Sample D also exhibited heterogeneous perovskite distribution, which led to micron-sized gaps, hole transport inside the titanium scaffold, and voids at the interface of the hole transporting materials and electrode. In the temperature range of 20–90 °C, the normalized photovoltaic conversion efficiency (NPCE, ~0.95) was almost stable in the case of Sample C (20 to 90 °C). In case of Sample A, the NPCE was around ~0.86 at 20 °C, and it was decreased below 0.8 at 90 °C. The NPCE of samples B and D was also decreased with an increase in temperature from 20 to 90 °C.

In the case of some fragile energy materials like perovskites, the NPCE depends on the stability of the nanomaterial with respect to the changes experienced during heating conditions. Fan et al. employed Protochips in situ heating and a gas-supplied TEM system to investigate the thermal degradation of a perovskite structure during its transition at 85 °C (Figure 2C) [51]. The methylammonium lead iodide (MAPBI_3_) perovskite structure underwent a layer-by-layer thermal degradation pathway, wherein at moderate heating (85 °C), the tetragonal crystalline structure of MAPbl_3_ was gradually converted to a layered structure associated with the trigonal structure. The layer-by-layer degradation initiated at the surface demonstrated the lowest energy barrier and allowed crystal transition. Both experimental and theoretical studies confirmed that encapsulation with two-dimensional (2D) layered materials (boron nitride flakes and graphene) suppressed the surface degradation of perovskites and improved their thermal stability. Furthermore, the development of perovskite/2D sheet heterostructures can be expected to deliver significant environmental stability and photoelectrical performance. Mechanistic insights gained from this study can be utilized for developing perovskites with high thermal stability. Similarly, in another case, Yang et al. attempted to study the perovskite degradation pathway using in situ TEM and EELS for monitoring the microstructural degradation of MAPbI_3_ films in ambient air (relative humidity = ~30%) and in an N_2_-filled glovebox (Figure 2D) [52]. They examined the effect of vacuum conditions on the stability of perovskites. In the N_2_-filled condition, the NPCE initially decreased to 0.7 within 15 days; however, it stabilized at around 0.8 after 30 days and remained almost the same even after 120 days. On the contrary, as a function of time, NPCE rapidly decreased in samples kept in air-filled ambient conditions and became nearly zero within 15 days. In the vacuum condition, the Pb particles found initially became absent on the second day. The results revealed that vacuum alone does not allow the degradation of perovskites. At high vacuum conditions, after 30 days, the degradation was not continued. Significant degradation was noticed when heating to ~50–60 °C under light illumination. The authors predicted that, in the regular operating conditions of the solar cell (50–60 °C), provided with light exposure, heat seems to play a greater role in the degradation. The role of electrons and holes generated upon solar light illumination was not considered as the driving force that promotes the degradation. However, they did not have evidence to exclude the effect of light on degradation. Instability at low temperatures limits commercial applications. It was found that when exposed to air at ambient conditions, methyl ammonium (CH_3_NH_3_) and the organic moiety in perovskites are more prone to be attacked by oxygen than the PbI_6_ component of perovskites. Therefore, a tailored and oxidation-resistant organic moiety is required to prevent the degradation of perovskites in ambient conditions.

### 3.2. In Situ Gas Phase/ETEM-Based Characterization

Most practical information and new insights are obtained from the in situ TEM observations, especially regarding gas–solid interactions which can alter the growth mechanism, functionality, and properties of energy storage and conversion materials [18,48,53,54,55]. Under a gaseous environment, a windowed gas cell chamber or differential gas pumping approach is usually followed to maintain typical control over high-pressure limits, composition, extreme thermal response conditions, specimen drifts, etc. Advanced designs of ETEM combined with STEM techniques are often equipped with closed gas cell-type windows, wherein the reaction environment with the desired volume and a confined specimen stage inside electron-transparent top chambers are trapped to allow controlled gas flow in the tiny sealed space of TEM equipment. Closed-cell type in situ ETEM studies are required for charactering nanoscale energy materials.

**Figure 3:**
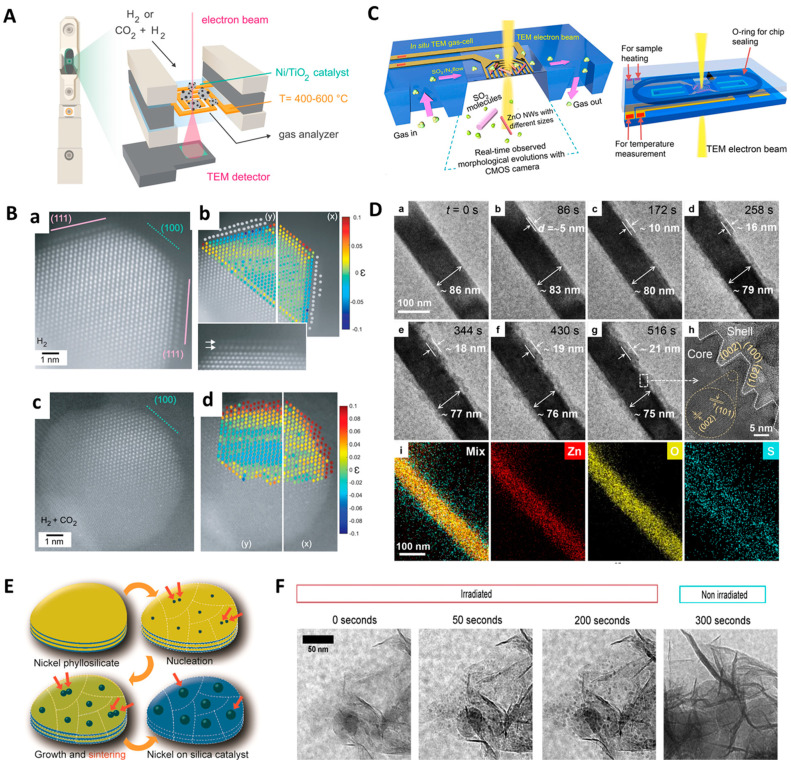
In situ gas-cell type TEM characterization for energy materials. (**A**) Schematic demonstration of operando ETEM equipped with windowed gas cell. (**B**) HAADF-STEM characterization depicting the atomic orientation of Ni/TiO_2_ (**a**) catalyst (exposed to H_2_) prepared under in situ conditions at 400 °C (white arrow: Ni NPs of TiO_2_ support, solid lines: TiO_x_-covered Ni atomic planes, and dashed lines: unoccupied facets), (**b**) strain maps showing atomic displacements, (**c**) catalyst (exposed to CO_2_:H_2_ (0.25 bar:0.75 bar) mixture) at 400 °C showing complete re-exposure of Ni and NPs restructuring, and (**d**) estimated atomic displacements/reorientations [59]. (**C**) Concept of loading ZnO nanowires onto SiN_x_ observing windows in a gas-cell setup with built-in MEMS chip (SO_2_ atm conditions). (**D**) In situ TEM imaging taken after exposure to SO_2_ gaseous conditions shows (**a**–**h**) the nanostructure’s morphological evolution, and (**i**) its corresponding EDS mapping [61]. (**E**) The concept of particle formation and growth observed during the reduction of nickel-phyllosilicate-based catalyst precursor was investigated in an in situ gaseous state. (**F**) In situ TEM images were observed at different time intervals during the reduction of nickel-phyllosilicates under 1 bar pressure (0.1 sccm gas flow rate at 425 °C) with an electron imaging dose of 30 e^−^·A^−2^·s^−1^ showing the nucleation and growth of nanoparticles only in the presence of the electron beam [63]. Figures reproduced with permission from [59,61,63].

Pre-treatment or high-temperature annealing conditions in supported metal catalysts often cause phase transition, restructuring, sintering, and structural oscillations which could eventually lead to deactivation of catalysts and cause poor selectivity [56,57,58]. Monai et al. carried out a combined microscopic and spectroscopic study to confirm the formation of suboxide overlayers on metal catalysts upon reduction [59]. Nickel-titanium dioxide (Ni/TiO_2_) catalyst used for industrial-scale CO and CO_2_ hydrogenation was studied. In situ analysis was performed after reduction at 400 and 600 °C. To track single atom-level information, a combination of in situ STEM, infrared spectroscopy, near-ambient pressure X-ray photoelectron spectroscopy, and DFT simulations was employed. A single Ni NP was tracked. Gaseous reactants and products from gas cells were tracked for the in situ spectroscopy studies. CH_4_ evolution was noticed upon introducing the CO_2_:H_2_ mixture. This confirmed that the catalyst is working for CO_2_ hydrogenation when observed by STEM. The process of suboxide overlayer formation is called strong metal–support interaction (SMSI). Carbon dioxide hydrogenation conditions removed the TiOx formed the surface of Ni/TiO_2_ during reduction (at 400 °C). After 600 °C reduction, carbon dioxide hydrogenation allowed only a partial re-exposure of nickel and formed TiOx at the interfacial sites. This provided a carbon species reservoir and favored C–C coupling. A partial TiOx removal and restructuring can occur even at low temperatures (200 °C); this led to the re-exposure of catalytically active Ni surface (Figure 3A,B). The outcome of this study challenges the conventional understanding of SMSI and requisites single particle-level studies for evaluating the structure–activity relationships. Another interesting study of nanocatalysts on support material was carried out by Epicier et al. using in situ ETEM to study the mechanism of controlling Pd particle genesis (size: ~5 nm) and its catalytic activity on δ-alumina support [60]. Formation of Pd NPs proceeded at 150–200 °C (6–10 mbar pressure) via spontaneous reduction of PdO. The number of Pd NPs formed in various environments (O_2_, air, and H_2_) was assessed. The oxidation state of Pd NPs, crystalline structure, and electron tomography were also given importance. Electronic tomography offers three-dimensional (3D) tracking in real time. Tracking experiments confirmed that Oswald ripening is a main cause for increasing the size of Pd NPs. To a certain extent, direct coalescence also contributed to an increase in particle size. Under the O_2_ atmosphere, new particle generation was also noticed during in situ calcination. Fast acquisition (a few seconds) in ETEM was largely supported for this study, especially for 3D tomography.

In some cases, 1D nanostructures were used to identify structural and phase transformations using gas-cell type in situ ETEM [61,62]. Generally, a cobalt catalyst is used in the growth of single-walled carbon nanotubes (SWCNTs). During SWCNT growth, Co undergoes phase and structural transitions, which cause Co transition across different inactive, active, and deactivated phases. Chao et al. used ETEM as a synthesis platform for the catalytic growth of SWCNTs [62]. To determine the types of planes that support anchoring or liftoff of SWCNTs from Co catalyst, the structure was analyzed using Miller indices. A density functional theory (DFT) model was used to study the interaction between different faceted planes of catalyst and graphene structure. In precise terms, SWCNTs’ growth and termination were directly proportional to the faceted planes and dominant phase of SWCNTs. The carbon-rich Co_2_C phase was observed to be either inactive or deactivated. In case of the Fe catalyst, the carbon-rich phase was reported to be favoring the growth. After deactivation, the associated faceted planes of the catalyst had no preference over interaction with SWCNTs. DFT models demonstrated that during anchoring and liftoff, the work of adhesion (W_adh_) of faceted planes in the proximity of the catalyst played a crucial role in determining the interfacial interactions between graphene sheets and catalyst. The W_adh_ of faceted planes adjacent to the catalyst NPs also played a huge role in the initiation and termination of SWCNTs’ growth. To prevent encapsulation of termination of growth, a difference in W_adh_ of faceted planes of catalyst is necessary. The difference in W_adh_ enables the propagation and liftoff of SWNTs. Since W_adh_ is relevant to all types of metallic catalysts, the authors suggest that the mechanisms can be applied to other systems involving the catalytic growth of SWCNTs. Furthermore, Wang et al. used in situ aberration-corrected TEM and quantitative structure−activity relationship (QSAR) models for studying the real-time sulfurization of ZnO nanowires (NWs) [61]. A gas cell was used to observe morphological changes in ZnO NWs (diameter = 100 and 500 nm) exposed to an SO_2_ environment (Figure 3C,D). The focus was also put on assessing the applicability of ZnO NWs in sensing and capturing SO_2_. Under the SO_2_ atmosphere, ZnO NWs (diameter ~ 100 nm), gradually transformed into a core−shell structure. In the outer surface of ZnO NWs, a ZnSO_3_ shell was formed. On the ZnO NPs with diameter of 500 nm, sparely distributed tiny NPs were found. This was attributed to the weak SO_2_–ZnO (gas–solid) interaction. Good reversibility (RSD = 1.5%, n = 3) was observed with the application potential of ZnO (100 and 500 nm) for the capture/storage of SO_2_. The sensing limit was 70 ppb. While thick ZnO NWs can be good SO_2_ sensors, thin ZnO NWs (100 nm) were found to be more suitable for SO_2_ capture/storage. The method established for the determination of quantitative solid−gas interactions demonstrates great potential for the synthesis and characterization of a variety of nanomaterials meant for SO_2_ capture and sensing.

Industrially relevant catalysts are usually formed by reduction, deposition, precipitation, and coprecipitation. There is a requirement for an activation step that converts soluble precursors (metal hydroxides, oxides, or silicates) to insoluble ones. High-temperature treatments (calcination, annealing, and reduction) convert these precursors into active catalysts. Reduction often converts metal oxide or silicate into metallic NPs. The reduction step is crucial for nucleation and the growth of NPs. Understanding the particle formation and growth mechanism is useful for controlling the performance of the resultant catalyst [64,65,66]. Ni NPs, a catalyst useful in the conversion of CO_2_ into fuel (methane), are prepared by reducing the nickel phyllosilicate catalyst precursor by 5% H_2_/Ar at elevated temperatures [67]. Turner et al. used in situ TEM for monitoring the formation of Ni NPs from nickel phyllosilicate (Figure 3E,F) [63]. The urea hydrolysis method was used to synthesize nickel phyllosilicate precursors via deposition precipitation. For in situ TEM studies, the calcined powder for nickel phyllosilicate precursor was dispersed in an ethanol drop and was cast on the chip and loaded in a Protochips Atmosphere system. The measurements were performed in a reducing condition (5% H_2_/Ar). The growth of Ni NPs occurred rapidly at 500 °C, 1 bar pressure, and 5% H_2_/Ar flow (0.1 sccm). Once the temperature reached 500 °C, the growth of Ni NPs was completed within 3–4 min, and the size reached was between 3.5 and 7 nm. The particle growth was followed by first-order kinetics. The growth of single Ni NPs was dominant. There was also a two-step particle growth, where two Ni NPs were nucleated and sintered together and their size was 5–7 nm, slightly bigger than a single Ni NP. Then, sufficiently grown particles were sintered together. Non-sintered Ni NPs remained smaller than sintered ones. Earlier nucleation was dominant and led to smaller Ni NPs, whereas late nucleation was rare and led to rapid growth. These findings supported the hypothesis that the size of the originating region eventually determines the size of Ni NPs. The size of the originating region is influenced by the synthesis of phyllosilicate such as defects, sheet thickness, and concentration. The size of the originating space needed to be evaluated in the near future.

Another interesting application of in situ ETEM involves the atomic-level CO interaction effect on the surface of bimetallic catalysts. Exposure to gas, heat, and liquid causes alloying, leaching, aggregation, and coarsening. These physiochemical changes had a detrimental effect on their catalytic activity. In platinum (Pt)-based bimetallic catalysts, the interaction between CO and catalysts has a profound impact on their efficiency and durability. Understanding the mechanism of structural modification induced by CO is important. Wang et al. investigated the dynamic change in the structure and composition of a PtPb@Pt nanoplate-type catalyst at the level of a single nanoplate [55]. Together with theoretical calculations (ab initio and DFT), in situ ETEM was used to execute the study in a CO gas environment. Evidence for atomic-scale discrete interactions between CO and the metal atoms was found at elevated temperatures. Pt atoms aggregated on the surface facets of the PtPb lattice. The Pt–CO complex propagated the cracking on the Pt surface in the PtPb alloys. Visual observation confirmed the separation of the Pt part as an island while Pb atoms stripped out of the alloy and formed Pb(CO)_4_, which is confirmed by resolving the lattice structure and atomic-scale imaging. These changes due to Pt–CO interactions led to the failure of the catalyst and require the development of CO-resistant PtPb catalysts. The strong metal–CO interactions caused the disintegration of PtPb@Pt nanoplates (NPLs) and eventual deactivation. The authors reported that the above effects can also be expected in other bimetallic catalysts including Pt.

## 4. In Situ Liquid-Cell TEM Setup for Characterizing Energy Materials

Monitoring a chemical reaction in liquid media is a challenging process since many random cohesive reactions can occur simultaneously in a liquid environment, which can lead to the scattering of electrons and cause improper characterization results. Hence, many customized liquid-cell designs with closed window columns under ultra-high vacuum conditions are utilized to control and monitor the activity of the liquid layer at the scale of tens and hundreds of nanometers. Liquid-cell TEM techniques are often applied to study the nucleation of nanoscale energy materials that are combined with electro-stripping/electro-plating procedures with the electrode systems. Such studies require a deep understanding of the electron beam effect on the specimen in a liquid-cell environment causing radiolysis with unpredicted chemical reactions when irradiated at 200–300 eV beam exposure conditions transferring energy to the liquid molecules [68,69,70,71,72,73,74].

**Figure 4:**
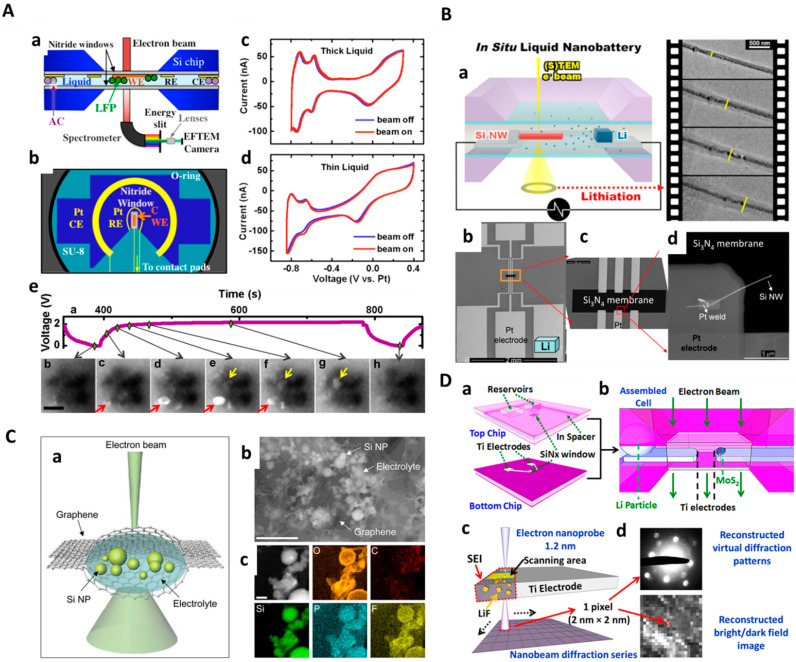
In situ liquid-cell/electrochemistry integrated TEM characterization for energy materials. (**A**) Schematic diagram showing a cross-sectional view of TEM holder, (**a**) conduit-like silicon nitride membranes encapsulating fluid layer and in situ electrochemical workstation, (**b**) system of three patterned electrodes with top chip, (**c**,**d**) electrochemical activity of Pt cyclic voltammetry (CV) with thick and thin liquid (~150 nm) layers, and (**e**) Temporal evolution (**a**–**h**) occurring in LiFePO_4_/FePO_4_ cluster during cycling (charge/discharge). Red, and yellow arrows indicate the propagation of delithiation in core–shell, and left to right pathways [75]. (**B**) Schematic representation of (**a**) in situ liquid-cell nanobattery setup analyzing the lithiation process, (**b**) SEM images of the electrochemically biased chip with (**b**) inner side, (**c**) its magnified view, and (**d**) Si NW electrode welded onto the Pt contact [76]. (**C**) Graphene liquid-cell (GLC) TEM illustration: (**a**) Si NPs were immersed in liquid electrolyte and placed between graphene layers in a sandwich structure, (**b**) the whole assembly was mounted on a holey amorphous carbon TEM grid (SEM image, scale bar: 1 μm), and (**c**) STEM mapping images of O, C, Si, P, and F in the GLC (scale bar: 100 nm) [77]. (**D**) Schematic diagram of in situ liquid-cell visualization of (**a**) MOS_2_ reaction on Ti electrodes, (**b**) assembled cell window area for capturing the dynamic lithiation/de-lithiation process, (**c**) schematic diagram for nanobeam diffraction characterization on the SEI layer or residual MoS_2_ reaction after the process, and (**d**) typical example showing bright-field or dark-field image reconstruction of the diffraction pattern [78]. Figures reproduced with permission from [75,76,77,78].

Intriguingly, Holtz et al. developed a quantitative electrochemical approach using liquid-cell TEM coupled with energy loss spectroscopy to imagine ion distribution at the microstructure level and observe local electronic structure change at the nanoscale during operation [75]. Specialized usage of valence energy-filtered TEM (EFTEM) with a flow cell helps in probing the low energy state (1–10 eV), which allows tracking charging-discharging of the battery as ions are transferred from the electrode to the electrolyte. An aqueous Li_2_SO_4_ (0.5 M) was used as an electrolyte. The electrode was biased with +2 V, and studies were performed as a function of time (0 to 1000 s). Based on an ab initio nonlinear response theory, they tracked the electronic structure of solvated ions and intercalated ions. In real time, while charging/discharging, the state of Li in the LiFePO_4_ electrode and aqueous electrolyte was determined with nanoscale resolution (Figure 4A). The transfer of Li between electrolyte and electrode and the charging dynamics were tracked. Under identical conditions, competing de-lithiation mechanisms were observed. In different particles, core−shell and anisotropic growth were observed simultaneously. EFTEM images showed gradual mass loss throughout the experiment. After five cycles, particle size was diminished by 20%. In control experiments, even before the exposure to the electron beam, fracturing and mass loss were observed all over the electrode surface. In real-time imaging, the particles disappeared from visibility after de-lithiation, and the formation of other de-lithiated regions was noticed. The de-lithiated region’s disappearance was attributed to the physical detachment of particles and floating into liquid electrolytes. Lattice strain caused during rapid cycling at 10 °C conditions made particles detach and float. The ability of the TEM liquid cell developed in this work can be effectively used to study the real-time dynamics of Li insertion and de-insertion and associated LiFePO_4_ degradation. Valuable insights gained from this work can be effectively utilized to understand the degradation pathway of electrode material in operando techniques.

Most of the Li metal battery applications require a detailed understanding with real-time observation of SEI formation and its growth kinetics. Meng Gu and coworkers demonstrated electrochemical liquid-cell operando TEM for the observation of lithiation and de-lithiation behavior of Si NW battery anodes (Figure 4B) [76]. For the real-time observation of structural and chemical evolution potentially by in situ TEM an open-cell configuration was used. Herein, the authors developed an operando TEM liquid cell for battery electrode studies and studied Si NW lithiation/de-lithiation behavior, revealing new insights. By using a novel in situ liquid-cell battery platform with a real electrolyte, they directly observed the lithiation and de-lithiation of submerged Si NW electrodes. This approach complements the open-cell method, enhancing the understanding of electrochemical processes. The open-cell technique provides detailed information on electrode composition and structural changes, while the liquid cell allows the study of realistic electrolyte–electrode interactions, including SEI formation and growth kinetics. Optimized cell development and electron dose calibrations can quantitatively measure the SEI layer and pave the way for advanced studies of SEI formation in working battery cells.

Recently, real-time analyses have provided valuable insights into the volume expansion of silicon (Si) nanomaterials during their electrochemical reactions with Li ions, aiding in the design of high-capacity Si anodes for LiBs. To further understand the critical first lithiation of Si in realistic liquid environments, Jong Min Yuk and co-workers used in situ graphene liquid-cell TEM (GLC-TEM) (Figure 4C) [77]. Electron-beam irradiation stimulated chemical lithiation, monitored by TEM in real time. Analyses revealed anisotropic volume expansion along the ⟨110⟩ directions during initial lithiation due to lower Li diffusion energy barriers. After this stage, Li diffusion became isotropic. This study demonstrates that in situ GLC-TEM is a valuable tool for understanding battery reactions of various active materials, especially during initial lithiation. Conventional TEM (JEM-3010, 300 kV, JEOL, Japan) with a fast-responding charge-coupled device (CCD) camera (SC200, Gatan, U.S.A.) was used for real-time observation of the lithiation process of Si NPs. The electron-beam dosage for imaging was set to 500–4000 pA/cm^2^ at an accelerating voltage of 300 kV to initiate the electron beam-induced chemical reaction. This study demonstrated that in situ GLC-TEM can observe real-time lithiation in Si NPs. Initial lithiation occurs preferentially along the ⟨110⟩ directions, but after this stage, Li diffusion becomes isotropic. This indicates that the rate-limiting diffusion barrier is at the Si–electrolyte interfaces, not within the Si crystal or between lithiated and de-lithiated regions. These insights can guide the design and large-scale synthesis of high-capacity Si NP anode materials.

Later, Zhiyuan and coworkers systematically studied the lithiation and de-lithiation of molybdenum sulfide (MoS_2_) nanosheets (NSs) in a LiPF_6_/EC/DEC electrolyte for LiBs using electrochemical liquid-cell TEM (Figure 4D) [78]. The electrochemical workstation was connected to the TEM holder for in situ cyclic voltammetry measurements. A nanobattery with an LiPF_6_/EC/DEC electrolyte and Li metal electrodes was used. They observed dynamic dissolution and deformation of MoS_2_ during charging (1.8–1.2 V), with irreversible decomposition into 5–10 nm NPs near 1.1 V. SEI formation on the titanium (Ti) anode was characterized, showing a composition of C, O, F, and LiF nanocrystals. However, some MoS_2_ NSs expanded and deformed without decomposing. An SEI layer formed on the Ti anode with LiF nanocrystals, but no passivation film formed on the MoS_2_ cathode. This study provides insights for improving battery design and the application of transition metal dichalcogenides in energy devices.

Overall, diverse morphologies such as Si NWs, Si NPs, and MoS_2_ NSs have been studied for their dynamic behavior transition within commercial battery electrolytes, revealing critical insights into structural and chemical changes during battery operation. Firstly, operando TEM liquid cells were equipped to study Si NW anodes, observing lithiation/de-lithiation behavior and providing valuable data on SEI formation and growth kinetics [76]. This method allows for the direct observation of submerged electrode materials, complementing traditional open-cell techniques. Secondly, graphene liquid-cell TEM was employed to monitor the initial lithiation of Si NPs, uncovering anisotropic volume expansion along specific crystallographic directions due to lower Li diffusion energy barriers [77]. This approach proved essential for understanding the lithiation mechanisms in realistic liquid environments. Finally, an electrochemical liquid-cell-integrated TEM setup was used for analyzing MoS_2_ NSs, capturing their dynamic dissolution, deformation, and irreversible decomposition during charging [78]. Also, they characterized the SEI layer on the Ti anode, identifying its composition and structure.

## 5. In Situ Light-Induced TEM Characterization for Energy Materials

Real-time observation of dynamic processes occurring in materials science is highly challenging. Explicitly, in situ microscopy equipped with sensors for multiple external stimuli such as strain (or pressure), temperature, and electromagnetic fields were mostly not extensively studied in terms of light–matter interactions [79,80,81,82,83]. Hence, significant differences between the effects of light and electrons have to be well understood to bring on the radiation techniques that can be coupled with the pre-existing in situ TEM. Light-induced reactions can offer a plethora of photochemical phenomena to which common electron imaging techniques are mostly insensitive. Atomic-scale in situ experiments are important for understanding the relationship between the structure and reactivity of catalysts used in photocatalysis and water splitting. The active form of catalyst emerges in reaction conditions. Light, as an unintentional source, was first applied in TEM studies (around 1984) along with other external stimuli like heating stages during in situ experiments with semiconductors like CdSe, CdS, ZnO, and CdTe [84]. Unexpectedly, researchers noted that some simultaneous transition states could occur during electron imaging in coherence with light beams or illuminators. Later, spectrofluorimetry [85] and optical fiber techniques enabled the use of specific light beam irradiation like ultraviolet (UV) rays for site-specific analysis in TEM samples [86], similar to the light microscopy concept in sub-nano-second temporal resolution [87].

**Figure 5:**
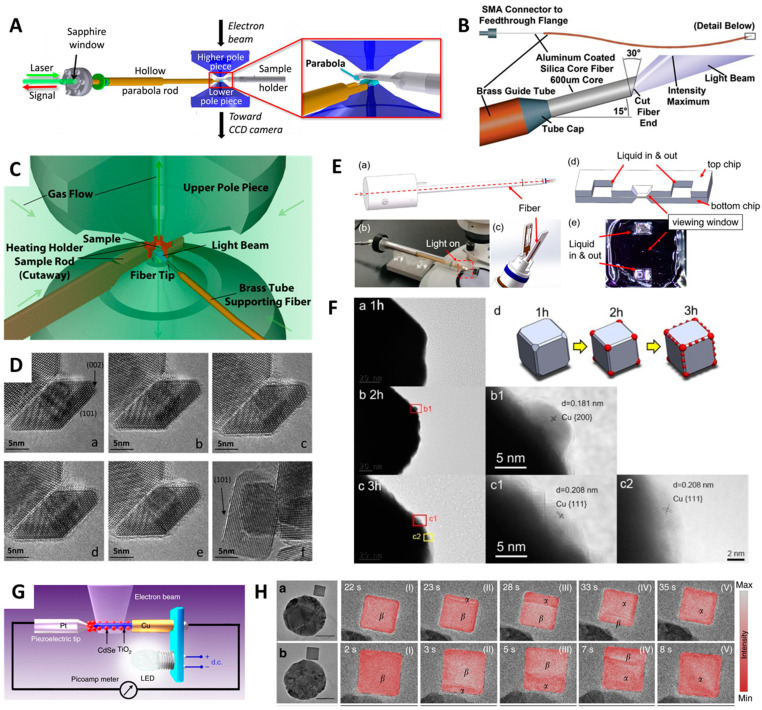
In situ light-induced TEM characterization of energy materials. (**A**) Schematic representation of integrated optical fiber setup with TEM holder [91]. (**B**) Customized fiber connection with cut fiber end projecting inside the microscope vacuum with a 15° angle to avoid optical loss, and the opposite angle cut by 30° to produce a beam that can illuminate the TEM sample [92]. (**C**) Schematic representation of in situ light-induced TEM with an integrated gas flow and heating controller inside the compact sample chamber. (**D**) In situ HRTEM imaging of anatase nanocrystals at 150 °C with/without 1 Torr water pressure: (**a**–**f**) diverse experimental conditions starting from no water to fresh water presence even after 40 h in a water/gas environment before exposure to the electron beam [93]. (**E**) TEM holder modification with liquid-cell chip arrangement. (**a**,**c**,**d**) schematic representations, (**b**) liquid cell, and (**e**) real photograph. (**F**) HRTEM analysis depicting transition in the morphology of Cu_2_O samples observed at different (**a**–**c**) irradiation time intervals such as 1 h, 2 h, 3 h, and (**d**) schematic showing its evolution over time [95]. (**G**) Schematic diagram displaying in situ fabrication of TiO_2_/CdSe nanowire QD solar cell integrated with LED and electrical measurement system in the STM-TEM [8]. (**H**) Light-induced rapid phase transformation (through *α* phase and hydrogen-rich *β* phase) reaction in the antenna–reactor configuration with illumination at 690 nm (scale bars: 50 nm) visualized over different short time intervals: (**a**,**b**) phase transformation at difffernt locations., in both cases (I–V) represents snapshots taken at different time (s) interval. Figures reproduced with permission from [8,91,92,93,95,97].

Generally, in photocatalysts, the in situ study must be conducted under appropriate light illumination [88,89,90]. Highly advanced optical systems integrating the environmental parameters with scanning TEM techniques known as ESTEM (Figure 5A) [91] possess the capability to deliver light precisely into the sample and generate/collect multiple spectroscopic signals during the dynamic transition conditions on the reactive media (sample). Miller et al. used a high-brightness broadband light source with optical fibers that can be precisely aligned with a fiber tip in an ETEM (Figure 5B) [92]. Zhang et al. studied the surface of TiO_2_ anatase NPs under the condition of vapor-phase water splitting (Figure 5C,D) [93]. Atomic resolution ETEM was used to study TiO_2_ particles subjected to water vapor pressure amounting to 1 Torr and light illumination. Broadband light (total intensity = 1430 mW/cm^2^) and UV–visible light absorbable by anatase (200–800 nm) having an intensity of 122 mW/cm^2^ were used as the illuminating light. Anatase powder dispersed on Stober silica spheres was drop-casted over Pt grids. Then, the Pt grid was attached to a Gatan hot stage in ETEM. In situ experiments were performed at 150 °C to simulate vapor-phase water splitting. The high-intensity broadband light source fitted inside the ETEM had an illumination intensity equal to 10 sun conditions (1430 mW/cm^2^). When exposed to light and water vapor, the crystalline surface of anatase was converted into a stable amorphous phase (1–2 monolayers). According to EELS and XPS studies, Ti in the amorphous layer had a +3 oxidation state. The thickness of the heavily hydroxylated amorphous layer was not increased over time. Under photocatalytic water splitting conditions, the amorphous layer was only present on the anatase surface. The same change was noticed when the anatase layer was subjected to electron irradiation and the energy absorbed was also constant. Direct photoreaction occurred between the top 1–2 monolayers in anatase crystal and adsorbed water caused them to become highly hydroxylated and amorphous. Furthermore, Weng et al. deciphered an interesting fusion mechanism for the coexistence of Au clusters on TiO_2_ NSs which caused migration, coalescence, and Ostwald ripening under UV irradiation in air [94], whereas, under inert N_2_ or vacuum conditions in UV light radiation, Au clusters depicted higher stability on the TiO_2_ surface in comparison to oxidizing environments.

Intriguingly, some semiconductor photocatalysts like Cu_2_O can undergo photo-degenerative transformations along the redox reaction mechanistic pathways. Such photocatalytic hydrogen evolution processes in these catalysts are of high industrial importance. Yu et al. studied the real-time observation of photocatalytic active sites in Cu_2_O, which self-reduce to nano-Cu on the particle surface [95]. Under light irradiation, the in situ formation of active sites on the Cu_2_O catalysts was observed with the help of a liquid-cell TEM setup. Self-reduction of Cu_2_O cubes to nano-Cu occurred when exposed to air, oxidizing the catalyst and decreasing the photocatalytic H_2_ evolution rate. This phenomenon of sequential self-reduction or in situ Cu_2_O structural change was characterized at different time intervals using a light-induced liquid cell-equipped TEM (Figure 5E,F). In another case, Cavalca et al. customized the TEM holder to observe the time-resolved systematic reduction of Cu_2_O to Cu under light irradiation of 405 nm coupled with water-vapor ETEM. Based on the ETEM observations, the photocatalytic degradation rate of Cu_2_O was closely assessed for nucleation reactions followed by EELS analysis [96].

Later, the idea of using a modified specimen holder with an LED source was highly beneficial for the analysis of photoelectric measurements of TiO_2_ decorated with CdSe quantum dots (QDs) (Figure 5G) [8]. Using the custom-manufactured in situ photoelectric STM-TEM probe, LED light-induced drifts were controlled reducing their movements during LED operation. This platform of detailed in situ photoelectrical studies in single TiO_2_-NW/CdSe-QD heterojunction solar cell provides valuable insights into the intrinsic interfacial properties at the nanoscale. Furthermore, a significant development in this light-induced research was realized after the observation of localized surface plasmon resonance (LSPR) effects using an aberration-corrected ETEM through the analysis of plasmon-dependent phase transformation of Pd NPs [97]. Utilizing controlled H_2_ pressure and temperature conditions, Pd NPs were subjected to accelerated phase transformations through *α*-phase and hydrogen-rich β-phase showing the LSPR-related phenomena at sub-2 nm resolution (Figure 5H). Later in 2021, the same group continued their research and published a paper demonstrating that tailored plasmonic excitation using optically excited plasmons could activate energetically unfavorable new nucleation sites accessible in plasmonic systems based on Au-PdH_x_ [98].

## 6. In Situ Cryo-TEM Characterization for Energy Materials

Cryo-TEM is one of the unique TEM techniques utilized when the samples are ultra-cooled to a very low temperature (particularly biological specimens) inside the microscope. This technique is highly useful for high-resolution characterization methods involving solid as well as liquid components including fragile interphases. Especially in the case of Li-metal batteries, vitrification of liquid electrolytes is needed to preserve the properties of the solid–electrolyte interphase in their native states, thus monitoring the morphological/structural mapping of the interfaces. Hence, Cryo-TEM is a useful tool for many catalysts that are adversely affected by the electron beam, such as radiation-sensitive energy materials, the SEI in rechargeable batteries, perovskite solar cells, fuel cells, and metal–organic framework (MOF) catalysts [3,4,7,99,100,101].

**Figure 6:**
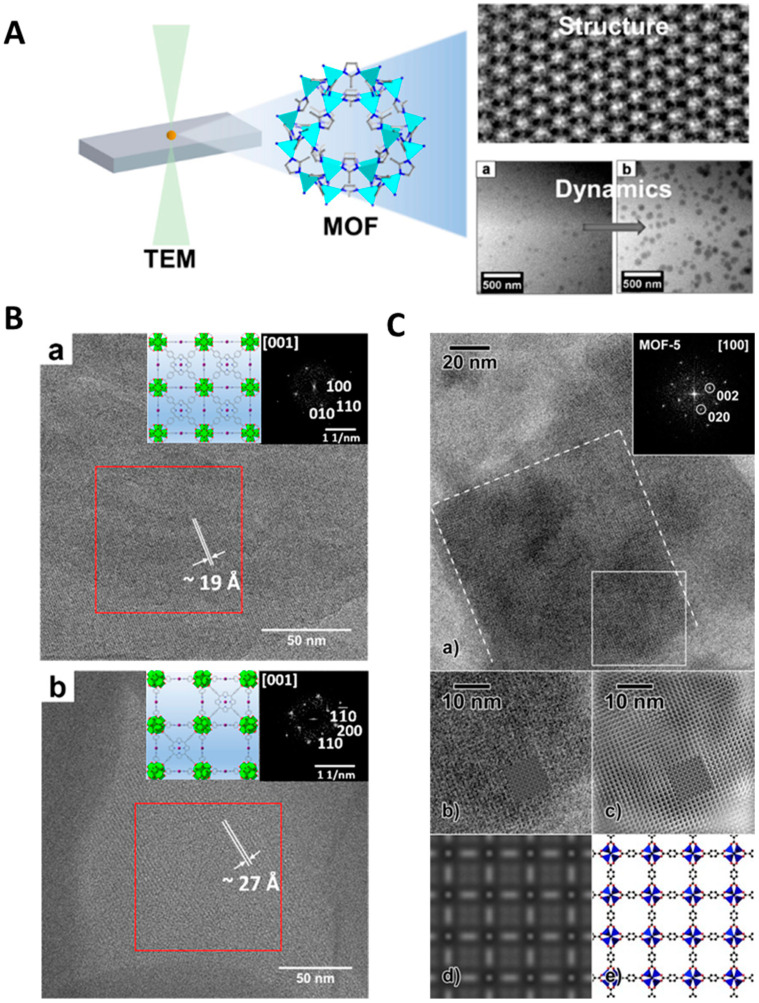
In situ Cryo-TEM characterization of MOF nanostructures. (**A**) Schematic representation of Cryo-TEM technique for MOF nanostructures for monitoring its dynamics under cryogenic conditions [100]. (**B**) HRTEM images of (**a**) MOF-525(Pt) and (**b**) PCN-224(Pt) (insets: FFT depicting the predominant lattice fringes) [102]. (**C**) HRTEM images of (**a**) intact MOF-5 crystals with inset FFT corresponding to [100] zone axis orientation as illustrated in (**b**); the selected inset from the filtered version (**c**) was simulated noise-free with (**d**) HRTEM image (thickness 15.5 nm, Δf = +12 nm, Cs +20 μm), and (**e**) illustrated structure of MOF-5 crystals (white stripes: terephthalate linker, grey color: pores) [103]. Figures reproduced with permission from [100,102,103].

In the case of radiation-sensitive energy materials like MOFs, specialized “low-dose” methods like Cryo-TEM techniques are necessary to characterize the innate structural backbone of polymer linkers, surface and interfacial structures, defects, and host–guest interactions with direct spatiotemporal observations (Figure 6A) [100]. Normal HRTEM methods can easily discern pore size and metal nodes in MOF nanostructures, whereas certain electron-dense metal nodes like structural organic linkers can reorient themselves under a high-dose electron beam. Specifically, in some MOF polymorphs MOF-525 ([Zr_6_O_4_(OH)_4_(TCPP-H_2_)_3_], TCPP-H_2_ = tetrakis(4-carboxyphenyl)porphyrin; ftw net), and PCN-224 ([Zr_6_O_4_(OH)_4_(TCPP-H_2_)_1.5_(H_2_O)_6_(OH)_6_]; she net) are a pair of MOFs that can be arranged in a same assembly of Zr_6_ nodes, but the porphyrin linkers can possess different orientations (Figure 6B) [102]. Even though these polymorphs exhibit alternate phase orientations, they can depict similar sorption behavior and X-ray diffraction (XRD) patterns during analysis. In addition, regular HRTEM characterization also cannot distinguish these structurally equivalent free base linkers (TCPP-H_2_); however, only their angstrom-level modifications in the lattice spacings of intrinsic porphyrin linkers with Pt unambiguously identified the difference. On the other hand, the pores of MOF-5 can be completely resolved under liquid N_2_ temperature conditions. Wiktor et al. analyzed the most intact <100> type zone axis, with predominant (100) surface facet orientation, by applying Fourier and low-pass filters for enhanced imaging of MOF-5 structural confirmations (Figure 6C) [103]. Hence, Cryo-TEM can play a crucial role in realizing transitions in radiation-sensitive materials like MOFs, polymers, etc. [7,99,100].

Interestingly, in the case of sensitive battery interfaces, monitoring the crystallographic transitions at the atomic-scale resolution of the solid–liquid interfaces is necessary to minimize the losses in the performance of rechargeable batteries [3,4,101,104].

**Figure 7:**
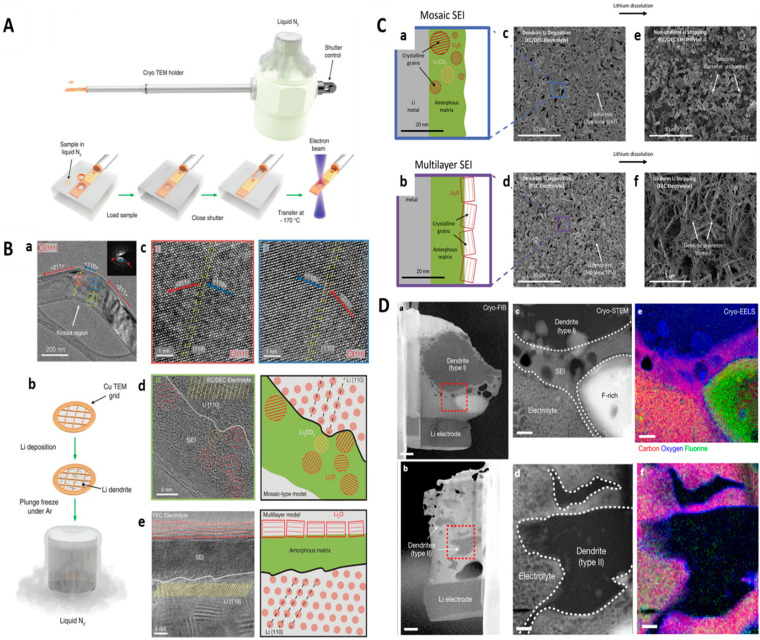
In situ Cryo-TEM characterization for SEI layer and Li metal interfaces in rechargeable batteries. (**A**) The method for preserving and stabilizing Li metal by Cryo-TEM whereby the specimen was placed onto the Cryo-TEM holder while maintaining cryogenic conditions, and during insertion of TEM column the temperature is maintained not above −170 °C. (**B**) Cryo-TEM observations (**a**) depicting kinked Li metal dendrite and SEI that changes from <211> to <110> and finally comes back to <211> growth lattice projection, (**b**) Li deposition on Cu TEM grid and storage under cryogenic conditions, (**c**) atomic-resolution images showing the transition from (i) <211> to <110> and (ii) <110> changes back to <211> lattice orientation, (**d**) mosaic model, and (**e**) multi-layer model of dendritic Li deposition on SEI under different carbonate electrolyte conditions [101]. (**C**) Detailed representation of Li dissolution under (**a**,**c**,**e**) mosaic SEI, and (**b**,**d**,**f**) multi-layer SEIs [3]. (**D**) Structural composition and elemental mapping of Li dendrites formed at the interface; the morphology was differentiated and analyzed under (**a**,**b**) Cryo-FIB, (**c**,**d**) Cryo-STEM, and (**e**,**f**) Cryo-EELS for type I dendrites and type II dendrites, respectively. Scale bar in (**a**,**b**), and (**c**–**f**) are equal to 1 µM, and 300 nm, respectively [4]. Figures reproduced with permission from [3,4,101].

For the first time, Li et al. preserved the chemical states of beam-sensitive battery materials under cryogenic conditions (Figure 7A) [101]. The atomic resolution of Li metal at the SEI was studied by preserving the Li metal under rapid freezing conditions. Electrochemically deposited Li metal dendrites (on a Cu TEM grid) were characterized in their native states after the first electrochemical deposition. Such stabilized dendrites tend to grow single-crystalline nanowires faceted along <111> most preferably, along with other <110> and <211> facets. Also, in carbonate-based electrolytes, they have observed that single-crystalline NWs often change their crystallographic growth directions (from <211> to <110>, and then back to their original facet) based on the kink formation sites (Figure 7B). Atomic interfaces between Li metal and the SEI layer are crucial to identify the efficacy of different electrolyte systems. Furthermore, the two distinct models (mosaic type and multi-layer type) of SEI nanostructures on the surface of Li metal dendrites revealed that the ordered multi-layer SEI nanostructures showed better mechanical durability than the random mosaic type. Notably, Li et al. studied the dynamic changes in the multilayer SEI nanostructures and demonstrated uniform Li stripping in electrochemical experiments which can be highly beneficial for high-energy batteries at the nanoscale [3] (Figure 7C). Interestingly, in another case, Wang et al. studied the electrochemically deposited Li (EDLi) in detail to explore its intrinsic physiochemical properties accompanying its SEIs [104]. The structural information revealed that EDLi evolution in the presence and absence of electrolyte additives was crucial at the nanoscale, to match its relationship with Coulombic efficiency that may cripple it due to excessive dendritic growth during lithiation chemistry. Engineering the surface properties of EDLi with additional functional metal ions to the electrolyte solution can slightly increase the efficiency of batteries under electrochemical cycling. Using Cryo-TEM, various influential factors like the electrolyte concentration, solvents, additives, and transition of nanoscale structures of the deposited Li metal and its SEIs can be more effectively optimized and studied in the near future. Meanwhile, Zachman et al. identified the coexistence of two dendrite types on the Li anode using a Cryo-FIB technique, such as type I and type II dendrites (Figure 7D) corresponding to partially oxidized Li metal and uniform Li hydride mapped through low-loss EELS spectroscopy [4]. SEI regions of cycled coin cell type arrangements were used in the two types, (i) a less curved shape with a larger contact area (in µm) (type I), (ii) a convoluted shape with a smaller contact area (in sub-µm) (type II), whereby the latter are more prone to disconnect from the electrode. A combination of Cryo-STEM EELS and FIB techniques provided access to nanoscale domains and compositions that are intact beneath the dendritic SEI and Li hydrides formed during electrochemical lithiation.

**Figure 8:**
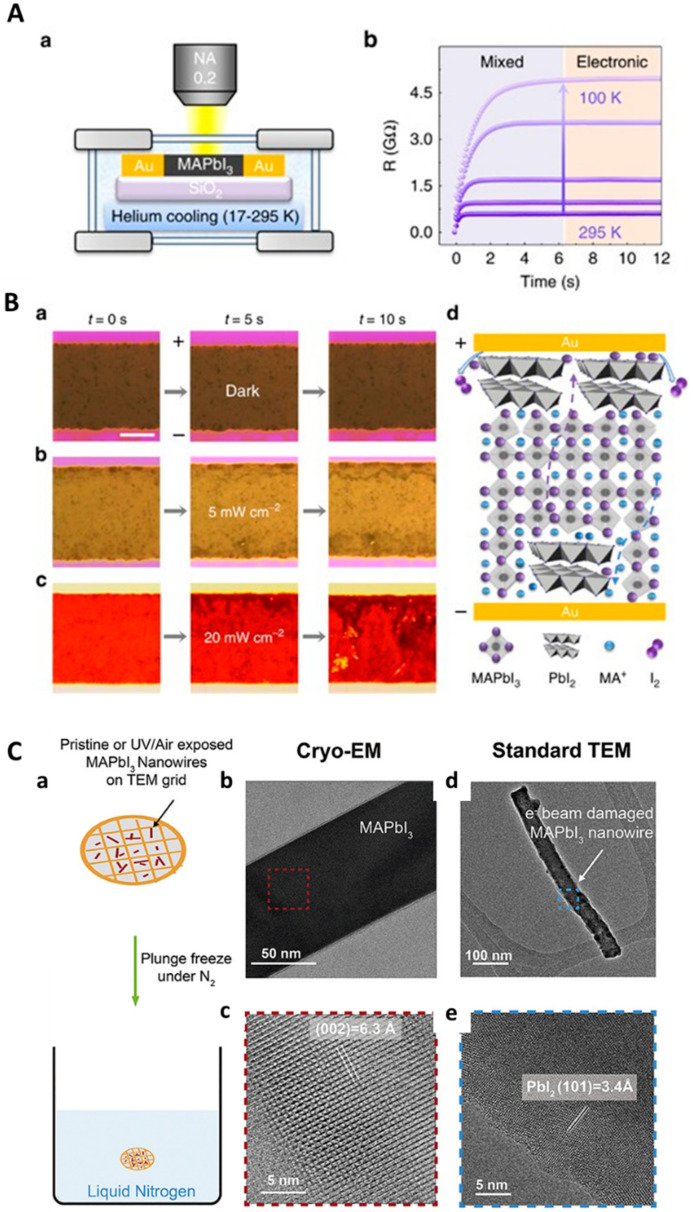
In situ Cryo-TEM characterization of electron-beam sensitive perovskites. (**A**) Schematic representation displaying (**a**) integrated galvanostatic and current-voltage measurements under Cryo-TEM conditions, and (**b**) five galvanostatic curves between mixed and only electronic states measured at the temperature range from 100 to 295 K. (**B**) Dynamic changes in optical images of perovskite films from (**a**–**c**) dark conditions to 20 mW cm^−2^ illumination, (**d**) schematic illustration depicting the migration of cations MA^+^ and anions I^−^ through the film [105]. (**C**) Drop casting of (**a**) pristine or UV/moisture-exposed perovskite nanowires onto the TEM grid in nitrogen glove box conditions, in comparison with using (**b**) Cryo-TEM showing preserved structures, (**c**) conventional TEM techniques showing electron beam-damaged structures with atomic-level resolution in lattice fringes, (**d**) TEM image of MAPI_3_ NWs after exposing to electron beam, and (**e**) decomposition of MAPI_3_ in to PBI_2_ [106]. Figures reproduced with permission from [105,106].

In the field of photovoltaic research, ABX_3_ perovskite structures are extremely prone to structural damage under high-voltage electron beam imaging. Cryogenic conditions can potentially suppress ion migration in such perovskites, where such ion migration triggered by an electron beam results in mixed electronic and ionic conductance, resulting in more difficulty in quantifying the composition. Zhao et al. combined the typical galvanostatic and I–V hysteresis to quantitatively demonstrate light-dependent ionic transportation in MAPbI_3_ perovskites by reducing the activation energy of cation/anion pairs I^−^/MA^+^ and H^+^ [105]. Due to the different responses of ionic and electronic transport to low temperatures, it became possible to distinguish between ionic relaxation and electronic resistance. When perovskite films were tested across a temperature range of 17–295 K and under light intensities from 0 to 20 mW cm^−2^, it was found that illumination decreased the activation energy for ionic transport by about a factor of five (Figure 8A,B). Additionally, employing cryogenic temperature-assisted characterization has broadened the understanding of perovskite materials. Similarly, Li et al. developed an interesting protocol with MAPbI_3_ NWs maintaining a critical electron dose of 12 e^−^/Å^2^ at −175 °C, resolving [PbI_6_]^4−^ octahedral and MA^+^ molecule columns at a spatial resolution limit of 1.49 Å [106] (Figure 8C). They discovered that a short UV illumination causes lead iodide NPs to precipitate on the surface of MAPbI_3_ NWs and causes a change in the surface roughness after 10 s of exposure to moisture. Also, under long-time UV illumination (1 h), the MAPbI_3_ NW structure does not decompose. This phenomenon was undetectable using XRD characterization, while the Cryo-TEM was delicate enough to monitor such crucial structural change at atomic-level resolution.

In case of fuel cell applications, the high-temperature operation capability of polymer electrolyte membranes in fuel cells favors water management and easy integration into high-purity fuel processing devices. Proton transport in typical poly(benzimidazole) (PBI) membrane fuel cells are unstable above 160 °C. With the combination of other tools, Lee et al. used Cryo-TEM to study the stability of a polymer electrolyte membrane (PEM) fuel cell made using *para*-PBI and cerium hydrogen phosphate (CeHP) [107]. During thermal cycling, maximum power density was reached at 250 °C (in dry H_2_/O_2_) with negligible degradation even after 500 h. The maximum operation temperature was 300 °C. An interconnected and self-assembled network of well-dispersed echinoid-shaped CeHP particles allowed stable proton transport at high temperatures (above 200 °C), and outperformed PEMs based on conventional p-PBI and CeHP. Thus, Cryo-TEM studies were effectively applied for the development of high-temperature operable and commercially viable PEM fuel cells.

## 7. Future Prospects and Advancements in TEM Characterization Techniques for Energy Materials

Integrating gas and heat conditions in solid-state or liquid-state in situ TEM systems can reveal the material’s evolution (at micro or nanoscale) information over a large number of cycles and can predict its performance at the bulk scale. This phenomenon can directly be correlated with the performance of practical devices that are affected by various internal and external factors (material geometry and dimensions, cycling-induced thermal damage, catalyst aging, etc.), and needs to be addressed in future energy cell in situ TEM investigations.

In terms of the liquid phase reactions, there is a necessity to observe the direct growth kinetics of nanomaterials at the molecular or atomic level possessing control over the reaction pathway and dynamics. Intriguingly, in situ TEM techniques can be combined with STEM and EELS spectroscopy to examine the composition showing an ideal foundation for structural and chemical analysis at the nanoscale. However, controlling the thickness of the liquid-cell chamber during in situ studies is highly desired to have a clear idea of atomic-resolution investigation for many catalysts, particularly in the case of single-atom catalysts, atomically resolved high-entropy alloys, etc. Liquid-cell systems in TEM studies can also direct future research in the practical imaging of several biological materials and systems in their native state without any cellular-level disturbance.

From a futuristic point of view, light-induced in situ TEM could be extended toward observing more interesting reactive sites in the most promising photocatalytic systems like TiO_2_ [87,94,108] and ZnO [10,109,110]. Generally, heterogeneous catalysis process involving a dual state of gaseous and solid products during catalysis could be characterized using the combination of light-induced probes in liquid-cell TEM as well as gaseous ETEM conditions [111]. Advancements in pulsed laser technology may lead to intrinsic studies on single NP levels with observations ranging from single photon to multi-photon excitation processes improving the LSPR effects at the atomic scale [112]. In terms of material processing techniques, photothermal processes along with laser ablation, sintering, and welding in situ experiments [113] can be monitored in detail considering the quantum-mechanical interactions of light and electrons [114,115]. Using low-dose electron imaging techniques on photosensitive semiconductors [116] it is possible to visualize stage-wise transitions in such systems, allowing the possibility of electron imaging even in some delicate micro-organisms in real time without reaching their lethal conditions [117].

With the ongoing development of cryogenic techniques, it is expected that further insights into perovskite materials will be achieved. Probably, the next big development in Cryo-TEM-based in situ characterization techniques would be the real-time analysis coupled with Cryo-FIB modules monitoring the functioning of perovskite devices, radiation-sensitive MOF structures and other energy materials, fragile polymer nanostructures, and SEIs with high-energy batteries [3,4,7,99,100,101]. This will allow researchers to unlock several direct observations on the degradation modes specific to operational conditions in devices, such as interfacial chemistry in Li metal-based batteries, halide segregation in solar cells, halide exchange in light emitting diodes (LEDs), and similar devices.

Some advanced combinatorial analyses such as vibrational spectroscopy in 4D/3D STEM-EELS analysis, 3D tomography with atomic-scale resolution, electron ptychography-based 3D structural analysis, phase-contrasting imaging, etc. [17,118,119,120] have progressed the spatial resolution in 2D and 3D analysis simultaneously. Such detailed investigations pave the way for multi-modal and multi-dimensional analysis of energy materials that can provide more real-time information about reaction dynamics.

## 8. Summary and Outlook

In summary, recent advancements in the state-of-the-art in situ TEM microscopic techniques highlight their unprecedented capabilities to explore the intrinsic synthetic and catalytic reaction mechanisms prevailing in advanced energy materials chemistry focused on more precise analysis at atomic-scale imaging resolutions. Progress in the field of in situ TEM setup at the nanoscale can offer unprecedented opportunities for the fundamental research exploration of energy materials. In situ TEM setup enables the practical exploration of real-time visualization of chemical reactions that can occur on the surface or at an interatomic level in various energy conversion and storage systems such as LiBs, fuel cells, perovskites, photocatalysts, etc. A future direction in energy materials chemistry could focus on the development of highly sophisticated in situ TEM investigations that are integrated with recently emerged imaging and spectroscopic techniques such as 4D-STEM and monochromated EELS. Such techniques play a critical role in gaining dynamic transitions from the catalysis perspective of energy materials. Furthermore, in the case of beam-sensitive energy materials, precise control over electron dose, line- or point-specific mapping, and spectral analysis techniques are powerful tools in combining fast-scan high-contrast imaging and machine learning-based signal enhancement in data analysis. These are some of the crucial factors in equipping advanced ultra-fast and multi-modal TEM systems that can improve the time and scan resolution of in situ imaging by several orders of magnitude. Atomic-scale tomography combined with machine-learning tools will create new opportunities, changing the perspective of data-acquisition algorithms that can simplify more complex atomic-scale information visualized under in situ microscopy, spectroscopy, and theoretical model-based calculations.

## Data Availability

Not applicable.
